# The causality between smoking and intervertebral disc degeneration mediated by IL-1β secreted by macrophage: A Mendelian randomization study

**DOI:** 10.1016/j.heliyon.2024.e37044

**Published:** 2024-08-28

**Authors:** Zhaopu Han, Yicheng Chen, Xiaojian Ye

**Affiliations:** Department of Orthopedics, Tongren Hospital, Shanghai Jiao Tong University School of Medicine, Shanghai, 200336, China

**Keywords:** Smoking, Intervertebral disc degeneration, Macrophage, Inflammatory factor, Mendelian randomization

## Abstract

There is still a lack of high-level evidence regarding the causal relationship between smoking and intervertebral disc degenerative diseases. This study utilized data from genome wide analysis studies and conducted two-sample Mendelian randomization analyses across multiple heterogeneous datasets. We evaluated the causal relationships between smoking behavior, serum inflammatory factors, serum chemokines, and intervertebral disc degeneration. Sensitivity analysis was performed to examine data heterogeneity and the pleiotropy of causal effects. The results indicated that smokers were liable to develop intervertebral disc degeneration (OR 1.770; 95 % CI, 1.519–2.064; p = 2.992 × 10–13), and long-term smoking behavior increased the risk of intervertebral disc degeneration (OR 1.715; 95 % CI 1.475–1.994; P = 2.220 × 10–12). Additionally, a causal relationship was confirmed between serum IL-1β level and intervertebral disc degeneration (OR 1.087; 95 % CI, 1.023–1.154; p = 0.007). The “smoking index” representing lifelong smoking habit was also found to be causally related to serum MCP-3 level(β = 0.292; SE = 0.093; p = 0.002). All of the causality mentioned above remained stable in sensitivity tests. Based on the analysis results and fundamental medicine theories around macrophage-induced inflammation in degenerative intervertebral discs, we have constructed a new mechanism that long-term smoking could induce an increase in serum MCP-3 level, promoting the gathering and activation of monocyte macrophages. Furthermore, the recruited macrophages led to an increase in local IL-1β within the intervertebral disc, ultimately exacerbating the process of intervertebral disc degeneration. What we have found is expected to accelerate the development of prevention and treatment of intervertebral disc degeneration.

With the ongoing development of aging society, degenerative diseases are increasingly receiving attention. As a common and typical degenerative disorder, intervertebral disc degeneration(IVDD) often leads to secondary clinical symptoms such as low back pain and sciatica, severely impacting the quality of life for patients and imposing a significant burden on the expenditure for social security [1]. To date, although the prevalence of IVDD is high, its risk factors and pathogenic mechanisms have not been fully explored. Traditionally, IVDD has been considered as a multifactorial disease influenced by both genetic and environmental regulation [2]. Smoking, as a contributing factor in the development of numerous chronic diseases, is also believed to mediate the chronic pathological changes of intervertebral discs by affecting bone mineral content, local blood supply, and nutrient metabolism. However, there is a lack of robust clinical evidence, and its specific mechanisms remain unclear [3,4]. Moreover, in recent years, many researchers have approached IVDD from the perspective of immune regulation, revealing a close association between metabolic disorders, inflammation, and the onset and progression of IVDD [5]. It has been reported that macrophages within locally degenerated intervertebral disc tissue can promote autophagy, senescence, and apoptosis of nucleus pulposus cells and annulus fibrosus cells by secreting cytokines such as tumor necrosis factor-alpha(TNF-α), interleukin-1 alpha/1 beta/2/4/6/10/17(IL-1α/1β/2/4/6/10/17), interferon-gamma(IFN-γ) [[6], [7], [8]].

To date, the potential relationship between smoking, inflammatory factors, and IVDD has been preliminarily confirmed in animal models and clinical studies. However, affected by factors including sample size, individual differences, and experimental protocols, the results of many related studies are inconsistent, and the causal relationships between various factors remain undefined. This is closely associated with the endogenous bias in previous studies, which is unable to exclude bidirectional causation and interference from confounding factors [9]. Additionally, owing to the macroscopic character of smoking behavior, there lacks research on whether it can mediate the occurrence of IVDD by acting on the serum level of inflammatory factors. Therefore, it is highly necessary to conduct a reliable causal inference study to determine the relationship between smoking, inflammatory factors, and IVDD.

Mendelian Randomization (MR) is a research paradigm that employs epidemiological approaches to assess causal relationships between risk factors and outcome indicators, which has been increasingly utilized to enhance the credibility of previous findings and provide big-data-based evidence for new clinical hypotheses in recent years [10]. As genetic variations are randomly assigned, MR can be adopted to minimize confounding bias by using single nucleotide polymorphisms(SNPs) as instrumental variables(IVs). In general, valid IVs must meet three hypotheses: (1) highly correlated with the risk factors also known as exposure; (2) acting on the outcome only through given exposure; (3) independent of all known confounding factors [9].

Given the limited research on the causal relationship among smoking behaviour, inflammatory cytokines and IVDD, in this study, we employed MR method to fill in the gap using various indicators. First, we analyzed and confirmed the impact of smoking on IVDD through univariate MR. After identifying the reliable cytokine IL-1β resulting in the occurrence of IVDD and failing to associate it with smoking behavior, we proceeded to select widely recognized serum chemokines that could enrich the monocyte-macrophage system and subsequently secrete pro-inflammatory factors. Then, MR analysis on the relationship between smoking behavior and potential chemokines were conducted. Ultimately, based on interrelated results, we have constructed a theoretical framework proposing that long-term smoking behavior mediates degenerative changes in intervertebral discs by inducing the secretion of IL-1β through chemotaxis of monocyte-macrophage cells. Our study provides a new mechanistic insight into the occurrence and development of IVDD.

## 1 Materials & methods

### 1.1 Study design

Firstly, a MR analysis was conducted to assess the causality between smoking behavior and IVDD from an epidemiological perspective. In the second step, appropriate cytokine markers related to IVDD were selected based on literature, subsequently with another MR analysis performed to determine the serum indicators that significantly affect the severity of IVDD. In the third step, we assessed whether there was a close relation between cytokines identified in last step and smoking behavior, trying to establish a theoretical framework for smoking behavior influencing IVDD occurrence and progression through the mediation of inflammatory factors. Then following closely, if a direct link between smoking behavior and serum factors could not be built, an attempt would be made to explore immune-regulatory cells mediating the pathological changes in IVDD. Univariate MR was employed to identify chemokines and cytokines that contributed to the recruitment and enrichment of local macrophages, thereby establishing a signaling pathway that long-term smoking would lead to an elevated chemokine level, which could activate the recruitment of monocytes to increase the secretion of local inflammatory factors, ultimately brought the exacerbation of IVDD. The genome-wide association study(GWAS) data used in this study were obtained from public databases, with informed consent and ethical approval [[11], [12], [13], [14], [15]].

### 1.2 GWAS data source

Given the population heterogeneity and data reliability, all MR analyses conducted in this study utilized data from individuals of European ancestry. The GWAS data for IVDD were sourced from the Finngen consortium, comprising 33,360 cases and 248,831 healthy controls. The diagnostic criteria for IVDD were ICD-10 M51, ICD-9 722, and ICD-8725 [11].

Regarding smoking behavior, we selected three variables from different perspectives to quantify the degree of severity. The GWAS data for “Smoking Initiation” and “Smoking Cessation” were sourced from the GSCAN study. For the former, there were 5994 cases and 2,663,035 controls. For the latter, there were 3160 cases and 1,144,112 controls [12]. Additionally, considering interfering factors such as smoking duration, intensity, and cessation, we utilized the variable named as “Smoking Index” constructed by Wootton et al. to comprehensively assess long-term smoking behavior. The original data for smoking index were obtained from the UK Biobank study, with 249,318 never-smokers, 164,649 individuals with a history of smoking, and 48,723 current smokers [13].

For serum inflammatory markers, we selected TNF-α, IL-1α, IL-1β, IL-2, IL-4, IL-6, IL-10, IL-17A, and IFN-γ from the INTERVAL cohort for evaluation. This cohort quantified 3622 kinds of plasma protein data of 3301 participants [14]. For monocyte-macrophage chemokines, we adopt indicators in the SCALLOP consortium for evaluation, including Monocyte Chemotactic Protein 1(MCP-1), Monocyte Chemotactic Protein 2(MCP-2), Monocyte Chemotactic Protein 3(MCP-3), and Macrophage Inflammatory Protein 1α(MIP-1α). This consortium integrated 90 plasma proteins from 13 cohorts, encompassing a total of 30,931 subjects [15].

All datasets listed above were analyzed as exposures or outcomes when performing MR, any of which shared an overlap rate with others less than 10 %. The information of included studies and consortia is presented in Supplementary Table 1.

### 1.3 IV selection

In terms of univariate MR conducted, we used the following two criteria for instrument variable selection: (1) SNP significantly correlated with the exposures(p < 5 × 10^−8^). (2) Linkage disequilibrium existed among index variables within 1 MB([LD] r^2^ < 0.001). Exceptionally, when selecting serum cytokines as exposures to analyze their causal relationship with the severity of IVDD, allowing for the fact that the exposure-related p-value threshold set as 5 × 10^^−8^ would give rise to available SNPs insufficiency, we adjusted it to 5 × 10^−6^ [10]. The F-statistic of each SNP was computed before causal analysis to exclude weak IV [16,17].

### 1.4 Causal and sensitivity analysis

**Fig. 1:**
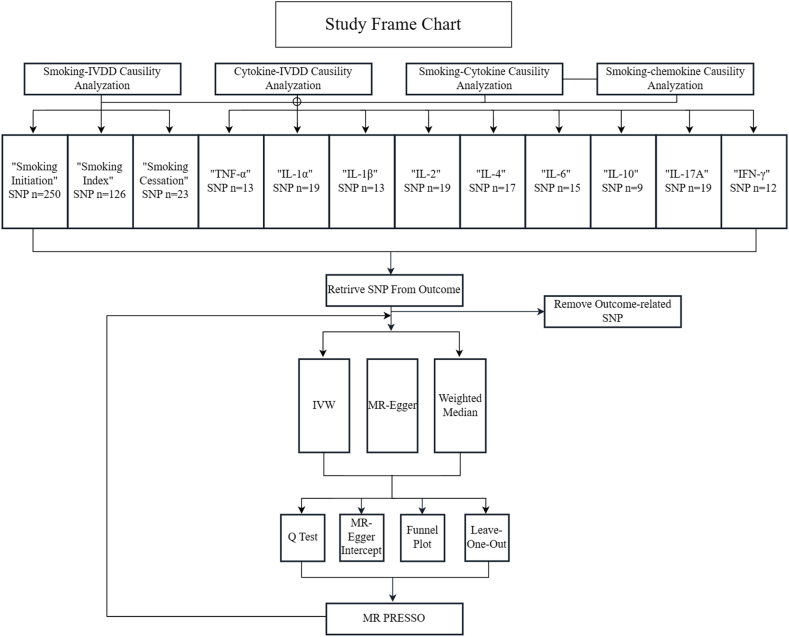
Roadmap for research. IVDD, intervertebral disc degeneration; TNF-α, tumor necrosis factor-alpha; IL-1α/1β/2/4/6/10/17A interleukin-1 alpha/1 beta/2/4/6/10/17A; IFN-γ, interferon-gamma.

We employed the inverse variance-weighted(IVW) method, the weighted median method, and the MR-Egger method, to assess the causal effects between different indicators. Since the MR Egger method was based on the premise that all SNPs participating in analysis exhibit pleiotropy in the absence of association with the exposure and the weighted median method allowed the presence of invalid instrumental variables with at least half of valid SNPs, we considered the IVW estimates as the primary results for causal inference, with the other two methods serving as references to test result sensitivity [10,18]. As for heterogeneity assessment, we utilized the Cochran Q test and the MR-PRESSO method. The former was applied to determine whether a random-effect or fixed-effect model should be selected for the IVW method in MR analysis, while the latter was employed to identify and correct for outliers, testing the consistency of causal effects. To examine the horizontal pleiotropy, we referred to the MR-Egger intercept test and leave-one-out analysis. And for the directional pleiotropy, we utilized funnel plots for visual inspection. The overall study framework is illustrated in Fig. 1.

### 1.5 Statistical tools

All analyses conducted in this study were performed via functions from the TwoSampleMR package (version 0.5.6) and MRPRESSO package (version 1.0) in the program R(version 4.2.2). For two-sided hypothesis testing, the threshold was set at 0.05.

## 2 Results

### 2.1 Causal relationships between smoking behaviour and IVDD

After filtering by GWAS p-value, LD clumping and F-statistic verifying, regarding the exposures “Smoking Initiation”, “Smoking Index”, and “Smoking Cessation”, we respectively identified 250, 126, and 23 SNPs as IVs, thereby satisfying the first hypothesis of MR research(Supplementary Table 2).

According to the IVW method, the results of MR indicated a causal relationship between smoking behaviour and IVDD, concretely the exposure “Smoking Initiation”(OR 1.770; 95%CI, 1.519–2.064; p = 2.992 × 10–13) and “Smoking Index”(OR 1.715; 95%CI 1.475–1.994; P = 2.220 × 10–12). However, the effect of “smoking cessation” for IVDD could not be confirmed(p = 0.808).

**Fig. 2:**
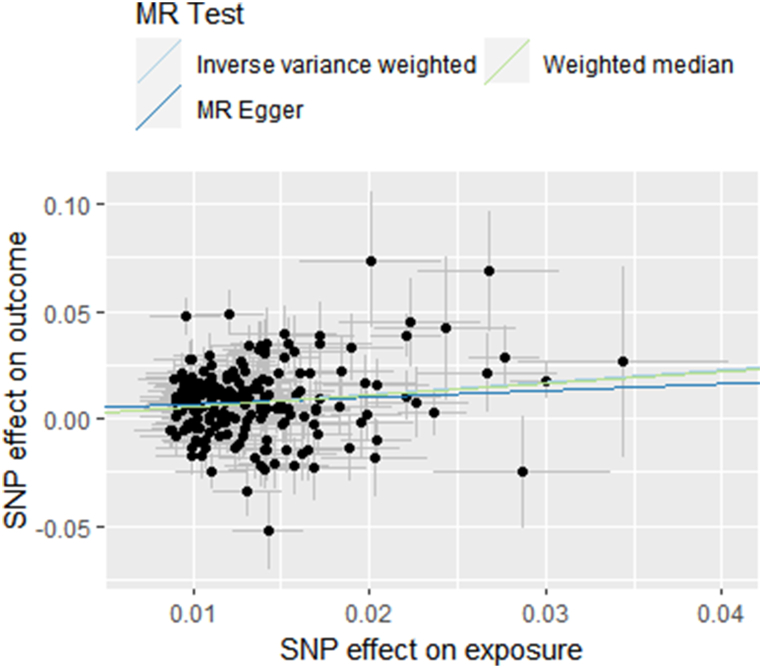
Scatter plot of the relationship between “Smoking Initiation” and IVDD using inverse-variance weighted, simple median, MR-Egger methods.

**Fig. 3:**
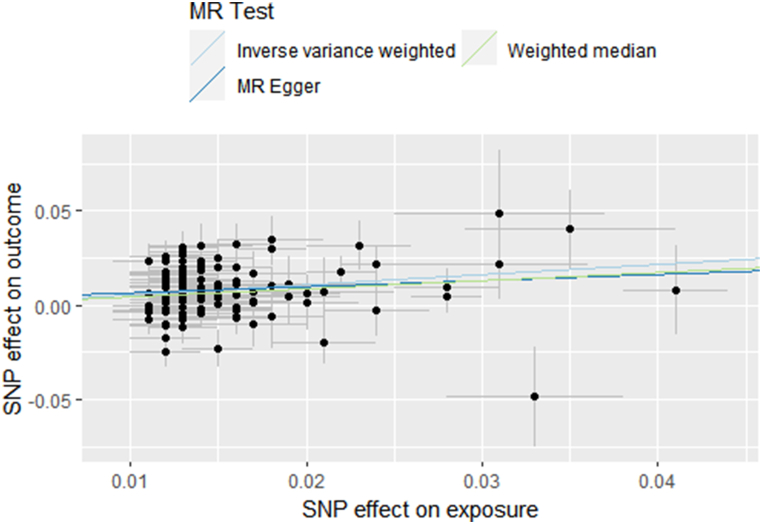
Scatter plot of the relationship between “Smoking Index” and IVDD using inverse-variance weighted, simple median, MR-Egger methods.

In terms of effect direction, the results drawn from the MR-Egger(“Smoking Initiation”-IVDD: OR 1.368; 95%CI, 0.770–2.432; p = 0.286. “Smoking Index”-IVDD: OR 1.389; 95%CI, 0.742–2.599; p = 0.306) and the weighted median(“Smoking Initiation”-IVDD: OR 1.730; 95%CI, 1.440–2.079; p = 4.623 × 10^−9^. “Smoking Index”-IVDD: OR 1.547; 95%CI, 1.282–1.867; p = 5.204 × 10^−6^) aligned with the IVW method(Fig. 2, Fig. 3). Considering that the assumptions of the MR-Egger method were too strict, it was acceptable that the significance level exceeded the threshold.

The MR-Egger intercept test showed that there was no significant difference between the intercepts and 0 for the relationship between “Smoking Initiation” or “Smoking Index” and IVDD, demonstrating the absence of significant horizontal pleiotropy within causal effects. Revealed by MR-PRESSO, three potential outliers causing horizontal pleiotropy were eliminated in “Smoking Initiation"-IVDD analysis. And the updated causal effects for all three analytical methods were consistent(IVW: OR 1.743; 95%CI, 1.508–2.015; p = 5.294 × 10^−14^. MR-Egger: OR 1.429; 95%CI, 0.832–2.454; p = 0.197. weighted median: OR 1.730; 95%CI, 1.433–2.089; p = 1.153 × 10^−8^). Similarly, one potential outlier were removed in “Smoking Index"-IVDD analysis, indicating robust results as before(IVW: OR 1.748; 95%CI, 1.510–2.023; p = 7.451 × 10^−14^. MR-Egger: OR 1.298; 95%CI, 0.708–2.380; p = 0.402. weighted median: OR 1.558; 95%CI, 1.299–1.869; p = 1.802 × 10^−6^). It could be seen intuitively through leave-one-out plots that any SNP removal did not affect the results significantly, suggesting the reliability of effects. Additionally, the funnel plots illustrated no obvious directional pleiotropy in these analyses(Supplementary Figs. 1 and 2).

### 2.2 Causal relationships between serum inflammatory cytokines and IVDD

With respect to the exposures TNF-α, IL-1α, IL-1β, IL-2, IL-4, IL-6, IL-10, IL-17A and IFN-γ, we respectively identified 13, 19, 13, 19, 17, 15, 9, 19 and 12 SNPs as IVs(Supplementary Table 2). The selecting procedure was the same as described in **2.1**.

**Fig. 4:**
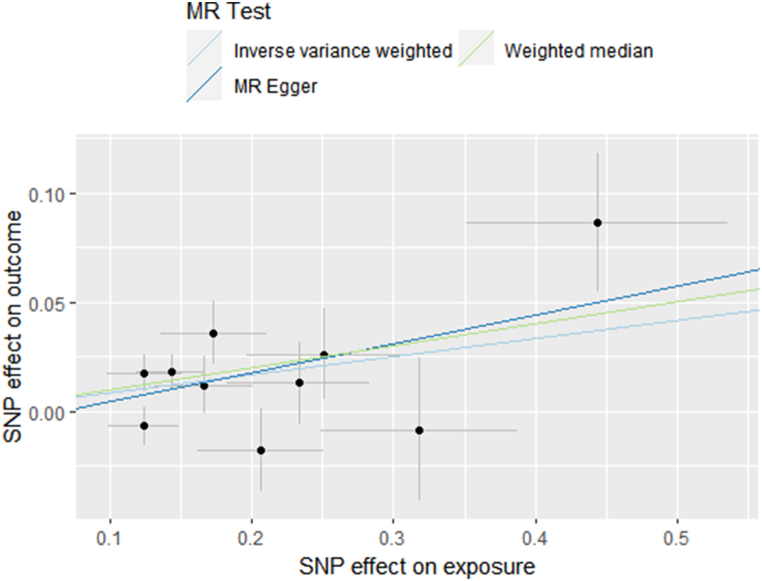
Scatter plot of the relationship between IL-1β and IVDD using inverse-variance weighted, simple median, MR-Egger methods.

The results of MR causal analysis using the IVW method indicated that all of the chosen cytokines had no significant causal effects on IVDD(p > 0.05). Heterogeneity was detected through Q-test, suggesting the presence of outlier data that potentially influenced the MR results. Therefore, MR-PRESSO was employed to remove outliers, and subsequent pairwise analyses between serum inflammatory cytokines and IVDD were conducted. The updated causal analysis results using the IVW method suggested a significant causal relationship between serum IL-1β level and the occurrence of IVDD(OR 1.087; 95 % CI, 1.023–1.154; p = 0.007), while other factors’ causality with IVDD were not confirmed(p > 0.05). As for IL-1β, the results obtained from the MR-Egger(OR 1.141; 95 % CI, 0.941–1.383; p = 0.217) and weighted median method(OR 1.106; 95 % CI, 1.030–1.187; p = 0.005) were consistent with the IVW estimate in terms of the effect direction(Fig. 4).

The MR-Egger intercept test suggested no implication of significant horizontal pleiotropy in the study of the causality between IL-1β and IVDD. And the leave-one-out plot and funnel plot demonstrated the robustness furthermore(Supplementary Fig. 3).

### 2.3 Causal relationships between smoking behaviour and serum inflammatory cytokines

The selection of IVs for smoking-related indicators was as previously described(Supplementary Table 2). Since the causal relationship between “Smoking Cessation” and IVDD had not been confirmed, it would no longer participate in univariate MR with potential intermediate factors.

All three methods of MR failed to confirm the causality between “Smoking Initiation” or “Smoking Index” and IL-1β. And we did not identify any abnormal SNP for removal via MR-PRESSO. These results implied that smoking behavior could not directly mediate IVDD by increasing serum IL-1β level. Since we had already demonstrated through univariate MR analysis that incremental serum IL-1β affected positively the occurrence of IVDD, combined with theoretical considerations of human blood circulation and drug metabolism, we justified in believing that elevating local IL-1β level in intervertebral disc tissue was able to promote the occurrence of IVDD. Moreover, according to literature, the local accumulation of monocyte-macrophage cells was critical for exacerbating inflammatory reactions in tissues. Therefore, we assumed that smoking behavior might induce the aggregation of macrophages in intervertebral disc tissue, leading to the secretion of IL-1β and further to mediate the development of IVDD. The validation of this hypothesis would be accomplished through MR analyses of “Smoking Initiation” and “Smoking Index” with serum macrophage chemokines.

### 2.4 Causal relationships between smoking behaviour and monocyte-macrophage chemokines

We adopted independent SNPs of “Smoking Initiation” and “Smoking Index” identified before(Supplementary Table 2). A causality between “Smoking Index” and serum MCP-3 were found using IVW method(β = 0.292; SE = 0.093; p = 0.002). Though, “Smoking Initiation” was chemokine-irrelevant, and the rest chemokines showed no association with smoking behavior(p > 0.05).

**Fig. 5:**
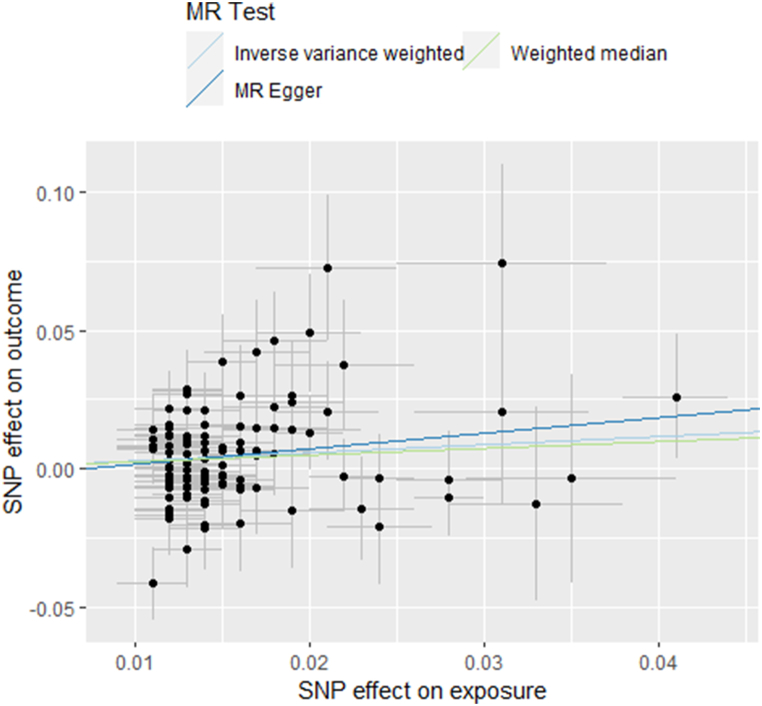
Scatter plot of the relationship between “Smoking Index” and MCP-3 using inverse-variance weighted, simple median, MR-Egger methods.

The effect direction of “Smoking Index"-MCP-3 with MR-Egger and weighted median method coincided with IVW's(MR-Egger: β = 0.562; SE = 0.368; p = 0.129. weighted median: β = 0.247; SE = 0.129; p = 0.056)(Fig. 5). Given the strict assumption of MR-Egger, as well as the approximate effect size and the slightly higher p-value under weighted median method, we still had enough faith in the causal relationship between “Smoking Index” and serum MCP-3.

Subsequently, no significant interference of horizontal pleiotropy was identified by MR-Egger intercept test and MR-PRESSO. And the leave-one-out plot and funnel plot confirmed that “Smoking Index” had a reliable impact on serum MCP-3 level(Supplementary Fig. 4).

**Fig. 6:**
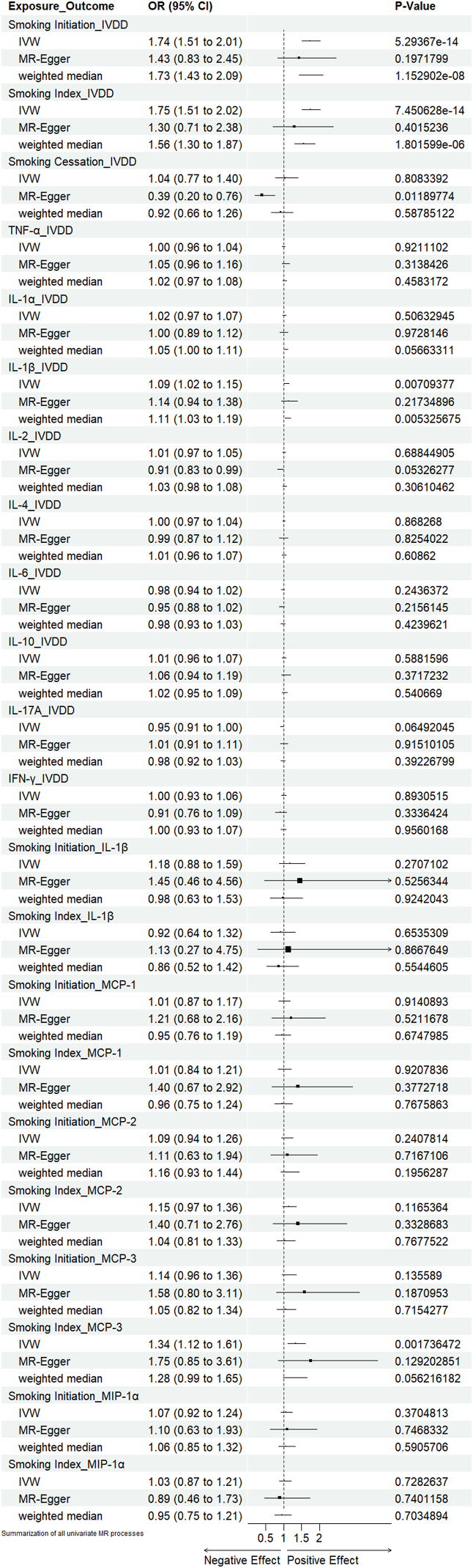
Forest plot of all included MR objects using inverse-variance weighted, simple median, MR-Egger methods. IVDD, intervertebral disc degeneration; TNF-α, tumor necrosis factor-alpha; IL-1α/1β/2/4/6/10/17A, interleukin-1 alpha/1 beta/2/4/6/10/17A; IFN-γ, interferon-gamma; MCP-1/2/3, Monocyte Chemotactic Protein 1/2/3; MIP-1α, Macrophage Inflammatory Protein 1α.

To summarize the overall process and give an intuitive illustration of the causal effect, we plotted a forest graph for all included exposure-outcome pairs after MR-PRESSO implementation(Fig. 6).

## 3 Discussion

In recent years, with the rapid development of big data and omics technologies, an increasing number of factors related to the occurrence and exacerbation of IVDD have been gradually uncovered [19,20]. Among these, repeated confirmations of traditional macroscopic risk factors and novel discoveries of microscopic metabolites within the human body are of equal essence. However, most studies are limited to demonstrating the univariate impact on IVDD, with less consideration given to connecting verified conclusions to construct a comprehensive chain of evidence regarding the entire pathological process of IVDD. Addressing this research gap, we started with the classic risk factor “smoking” and subsequently selected appropriate serum inflammatory cytokines and chemokines as intermediate factors, hoping to find out a potential pathway of IVDD occurrence.

Through this study, we further confirme that smoking behaviour is a significant hazard for IVDD. And the probability of IVDD occurrence increased with the advance of smoking duration and intensity. Additionally, smoking cessation is irrelevant with prevalence, suggesting the irreversibility of intervertebral disc degeneration. By introducing inflammatory or chemotactic factors, we demonstrate that an increase in serum IL-1β level can mediate the occurrence of IVDD, and long-term smoking can lead to an elevation in serum MCP-3 level. Based on these results, we propose that the effect of smoking on IVDD may be transmitted through the change of serum MCP-3, which recruits monocyte-macrophage cells from the circulatory system to accumulate in the intervertebral disc, following with IL-1β secreted to mediate the occurrence of IVDD.

The analysis is consistent with many previous studies in conclusion. As for the impact of smoking behavior on IVDD, numerous observational studies and systematic reviews have identified smoking as a key risk factor [4,21,22]. In particular, a recent MR study indicates a causal relationship between “smoking initiation” and thoracic/thoracolumbar/lumbosacral IVDD respectively, which can be regarded as refining of our first finding [23]. However, it is still unknown on how smoking mediates the occurrence of IVDD. Over the years, there have been successive reports on new mechanics. For instance, Jing et al. found that smoking induced apoptosis of anulus fibrosus(AF) cell through the local accumulation of cadmium [24]. Another research conducted by Tu et al. revealed that smoking, by motivating excessive activation of mast cell proteases, expedited the degradation of nucleus pulposus(NP) [25]. Such studies about IVDD mechanisms varied in celluar pathways, and yet they all explicitly pointed out the significance of inflammatory environment of degenerated disc tissue. While it has been universally acknowledged that the local inflammatory components always include immune cells such as macrophages, lymphocytes and dentritic cells, as well as inflammatory molecules like interferons, interleukins and chemokines. This suggests that smoking may promote the incidence rate of IVDD by changing local inflammatory response [26,27].

Among numerous immune components, macrophages have been consistently emphasized due to their role in bridging innate and adaptive immunity [28]. In terms of IVDD, with in-situ chronic inflammation being amplified and exogenous pro-inflammatory factors penetrating, locally resident or peripherally recruited macrophages can exacerbate the progression of degenerative changes through polarizing into pro-inflammatory phenotype to release inflammatory mediators including TNF-α and IL-1 [29]. Concretely, IL-1β is considered a key factor in IVDD deterioration [6]. In degenerated area, the balance between IL-1 and IL-1Ra is disrupted, as a result that the excess expression of IL-1β causes tissue degeneration by promoting inflammatory factor secretion, ECM degradation, and cell senescence [30]. It has been confirmed that the interaction of IL-1β with various inflammatory mediators can lead to the gathering of local reactive oxygen species(ROS) and mass expression of matrix metalloproteinases(MMPs) and a disintegrin and metalloproteinase with thrombospondins(ADAMTSs), accompanied by apoptosis, pyroptosis or ferroptosis of AF/NP cells [[31], [32], [33], [34], [35], [36]]. Moreover, targeted therapy against IL-1β has also shown promising results in inhibiting IVDD, further demonstrating the crucial role of IL-1β in IVDD pathogenesis [37,38]. Combining clues mentioned above, a pathway about IVDD occurrence and development, involving with successive roles of smoking, macrophage activation and IL-1β release, has been already uncovered.

MCP-3 is a member of the monocyte chemotactic protein family, first purified from human osteosarcoma cells(MG-63) in 1992 [39]. To date, most researchers have agreed on that MCP-3 can be secreted by various cells, including monocyte-macrophages, endothelial cells, and fibroblasts. Under physiological and various pathological conditions, MCP-3 acts on recruiting immune cells, stimulating specific protein expression and promoting angiogenesis [[40], [41], [42]]. Within degenerated disc, Kawaguchi et al. detected a growing expression of MCP-3 mRNA, suggesting that disc-derived MCP-3 may exacerbate IVDD by inducing macrophage infiltration [43]. As serum indicators are easy to detect and relatively stable, sometimes they can serve as substitutes for local cellular factors. In this study, serum MCP-3 was adopted as an analytical indicator to explore its potential connection with exposures. Although the molecular mechanisms by which long-term smoking habit mediates the elevation of MCP-3 levels require further investigation, the “exposure-outcome” evidence chain constructed above is sufficient to support the causality.

From the view of methodology, this study performed MR analyses, utilizing several public GWAS datasets to establish causal relationships between variables. The popular approach has been favored for its features of easily operable, cost-effective, and yielding high credibility in conclusions, addressing various shortcomings of traditional epidemiological study paradigm. Additionally, we employed multiple methods to test and interpret the heterogeneity and pleiotropy of MR results, aiming to enhance the reliability of the conclusion. However, it is essential to acknowledge a few limitations. Firstly, due to limited data sources, all data implicated in this study are derived exclusively from individuals of European ancestry. Therefore, the applicability of our findings to other ethnic populations remains to be explored. Besides, there is still no clear explanation for how long-term smoking increases the levels of chemokines, and the mechanisms involved require further basic research for clarification.

In summary, the study utilized MR analysis to confirm smoking behavior and serum IL-1β level had a causal impact on IVDD. Next, based on physiology theories and our findings that long-term smoking could elevate serum MCP-3 level, we constructed a new pathway of IVDD occurrence, noted as “Long-term Smoking→ Increased Serum MCP-3 Level→ Enrichment&Activation of Macrophages→ Local Elevation of IL-1β→ IVDD Exacerbation”. To our knowledge, this is the first MR study to speculate the mechanism by which smoking mediates the occurrence and development of IVDD, combing epidemiological research with fundamental theories. We hope that it will be helpful for the prevention and targeted treatment of IVDD, as well as methodological improvements in epidemiological research.

## Data availability statement

The datasets included in this study are publicly available and can be found in online repositories. Supplementary Table 1 presented the source information of GWAS data.

## Funding

This study was supported by Interdisciplinary Program of 10.13039/501100004921Shanghai Jiao Tong University (Grant No. YG2021ZD34).

## CRediT authorship contribution statement

**Zhaopu Han:** Conceptualization, Data curation, Formal analysis, Methodology, Software, Visualization, Writing – review & editing. **Yicheng Chen:** Data curation, Investigation, Methodology, Resources, Software, Visualization, Writing – original draft. **Xiaojian Ye:** Conceptualization, Formal analysis, Funding acquisition, Project administration, Supervision, Validation, Writing – review & editing.

## Declaration of competing interest

The authors declare the following financial interests/personal relationships which may be considered as potential competing interests:Xiaojian Ye reports financial support was provided by 10.13039/501100004921Shanghai Jiao Tong University. Xiaojian Ye reports a relationship with 10.13039/501100004921Shanghai Jiao Tong University that includes: employment, funding grants, and non-financial support. If there are other authors, they declare that they have no known competing financial interests or personal relationships that could have appeared to influence the work reported in this paper.
