# Supplementary material for: The causality between smoking and intervertebral disc degeneration mediated by IL-1β secreted by macrophage: A Mendelian randomization study
Source: Heliyon. 2024 Aug 28;10(17):e37044. doi: 10.1016/j.heliyon.2024.e37044 (PMC11402911; doi:10.1016/j.heliyon.2024.e37044)
Supplement: Multimedia component 1 [file mmc1.doc]

# Supplementary Figure

**
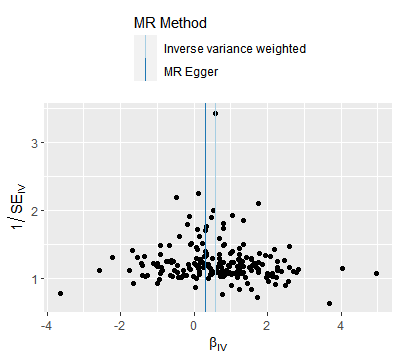

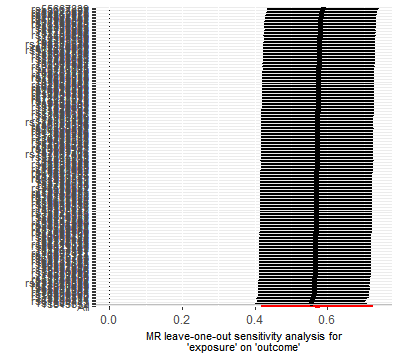
**

**Supplementary Figure 1: Funnel plot and leave-one-out analysis of the relationship between “Smoking Initiation” and IVDD**

**
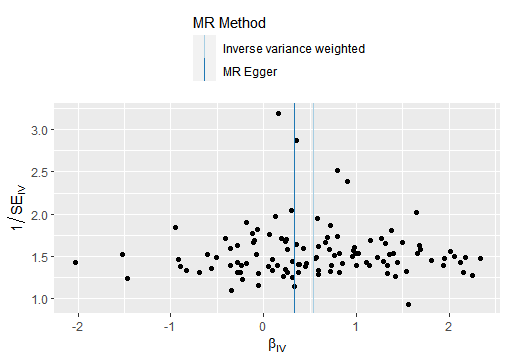

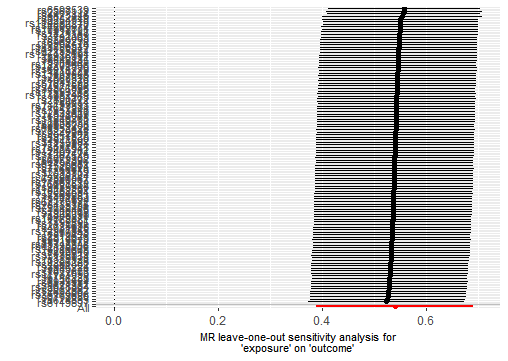
**

**Supplementary Figure 2: Funnel plot and leave-one-out analysis of the relationship between “Smoking Index” and IVDD**

**
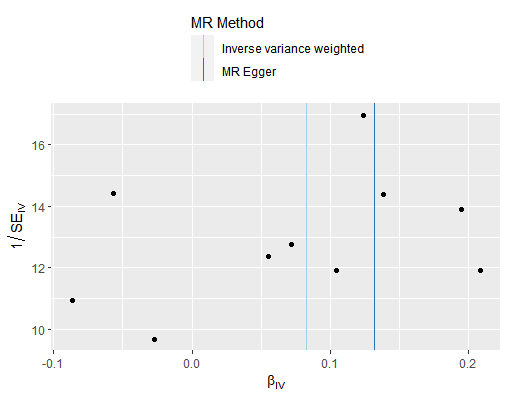

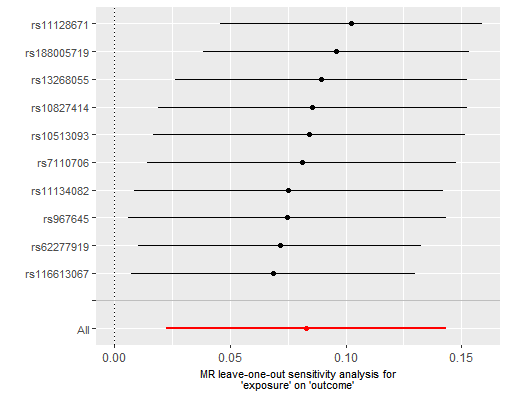
**

**Supplementary Figure 3: Funnel plot and leave-one-out analysis of the relationship between IL-1****β and IVDD**

**
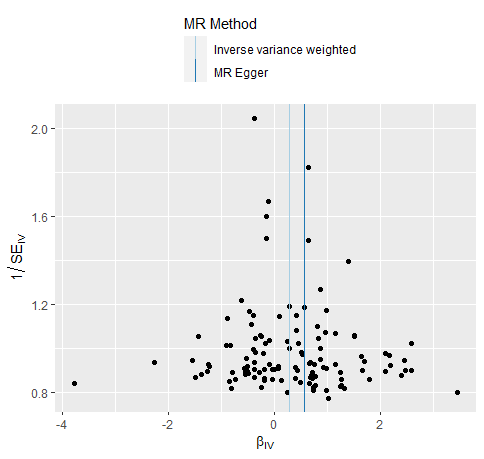

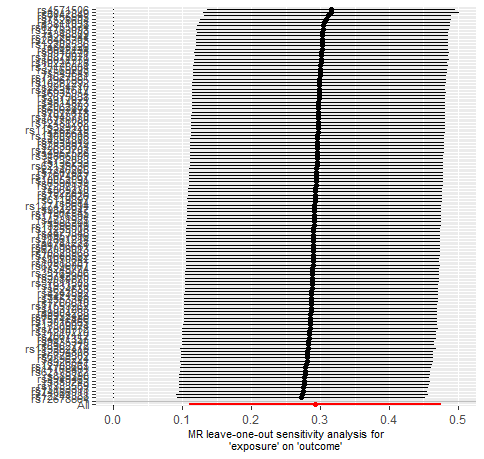
**

**Supplementary Figure 4: Funnel plot and leave-one-out analysis of the relationship between “Smoking Index” and MCP-3**
